# The psychosocial rehabilitation of the offending psychiatric patients: Looking the good practices

**DOI:** 10.1192/j.eurpsy.2021.2090

**Published:** 2021-08-13

**Authors:** A. Riolo, C. Battiston, A. Lusina, C. Sindici, U. Albert

**Affiliations:** 1 Department Of Mental Health, ASUGI, Trieste, Italy; 2 Department Of Mental Health, University of Trieste, Trieste, Italy

**Keywords:** Offending psychiatric patient, Psychoeducation, Psychosocial rehabilitation, Law

## Abstract

**Introduction:**

The Italian law 81/2014 has given a strong push to the design of therapeutic-rehabilitative paths for psychiatric patients who are offenders. This innovation requires a constant organizational effort on the part of mental health services to enforce the law. The rehabilitation team is represented by different professionals like psychiatrists, psychologists, nurses, psychiatric rehabilitation technicians, educators, social workers and others. They must be able to work in an integrated way among them and with private social sector.

**Objectives:**

It is in our interest to reach an agreement between different professionals working in the rehabilitation-forensic field about good practices.

**Methods:**

We have prepared a survey to identify good practices in the field of psychosocial rehabilitation of the offender psychiatric patient, involving different professionals who have expertise.

**Results:**

This audit revealed, in everybody’s opinion, that these offending citizens have received a security measure capable of having greater control over their actions in a therapeutic-rehabilitative perspective but it is fundamental to educate them also to exercise their own safety for a social shared culture. Ensuring the safety of the offender during the therapeutic-rehabilitative path is as important as responding to a society’s need for social security.

**Conclusions:**

Satisfying a society’s need for security, established by the Judge and the Law, all this cannot separated from the active exercise of security of the offending psychiatric patient towards himself, through psychoeducation. The safety towards others and towards oneself can constitute a good practice in the field of psychosocial rehabilitation.

**Disclosure:**

No significant relationships.

